# Detecting Gene-Environment Interaction for Maternal Exposures Using Case-Parent Trios Ascertained Through a Case With Non-Syndromic Orofacial Cleft

**DOI:** 10.3389/fcell.2021.621018

**Published:** 2021-04-16

**Authors:** Wanying Zhang, Sowmya Venkataraghavan, Jacqueline B. Hetmanski, Elizabeth J. Leslie, Mary L. Marazita, Eleanor Feingold, Seth M. Weinberg, Ingo Ruczinski, Margaret A. Taub, Alan F. Scott, Debashree Ray, Terri H. Beaty

**Affiliations:** ^1^Department of Epidemiology, School of Public Health, Johns Hopkins University, Baltimore, MD, United States; ^2^Department of Human Genetics, School of Medicine, Emory University, Atlanta, GA, United States; ^3^Center for Craniofacial and Dental Genetics, Department of Oral and Craniofacial Sciences, School of Dental Medicine and Clinical and Translational Science, School of Medicine, University of Pittsburgh, Pittsburgh, PA, United States; ^4^Department of Human Genetics, Graduate School of Public Health, University of Pittsburgh, Pittsburgh, PA, United States; ^5^Department of Biostatistics, School of Public Health, Johns Hopkins University, Baltimore, MD, United States; ^6^Department of Genetic Medicine, School of Medicine, Johns Hopkins University, Baltimore, MD, United States

**Keywords:** orofacial clefts, oral clefts, gene-environment interaction, case-parent trio design, genome-wide association study, maternal smoking, maternal vitamin supplementation

## Abstract

Two large studies of case–parent trios ascertained through a proband with a non-syndromic orofacial cleft (OFC, which includes cleft lip and palate, cleft lip alone, or cleft palate alone) were used to test for possible gene–environment (G × E) interaction between genome-wide markers (both observed and imputed) and self-reported maternal exposure to smoking, alcohol consumption, and multivitamin supplementation during pregnancy. The parent studies were as follows: GENEVA, which included 1,939 case–parent trios recruited largely through treatment centers in Europe, the United States, and Asia, and 1,443 case–parent trios from the Pittsburgh Orofacial Cleft Study (POFC) also ascertained through a proband with an OFC including three major racial/ethnic groups (European, Asian, and Latin American). Exposure rates to these environmental risk factors (maternal smoking, alcohol consumption, and multivitamin supplementation) varied across studies and among racial/ethnic groups, creating substantial differences in power to detect G × E interaction, but the trio design should minimize spurious results due to population stratification. The GENEVA and POFC studies were analyzed separately, and a meta-analysis was conducted across both studies to test for G × E interaction using the 2 df test of gene and G × E interaction and the 1 df test for G × E interaction alone. The 2 df test confirmed effects for several recognized risk genes, suggesting modest G × E effects. This analysis did reveal suggestive evidence for G × Vitamin interaction for *CASP9* on 1p36 located about 3 Mb from *PAX7*, a recognized risk gene. Several regions gave suggestive evidence of G × E interaction in the 1 df test. For example, for G × Smoking interaction, the 1 df test suggested markers in *MUSK* on 9q31.3 from meta-analysis. Markers near *SLCO3A1* also showed suggestive evidence in the 1 df test for G × Alcohol interaction, and rs41117 near *RETREG1* (a.k.a. *FAM134B*) also gave suggestive significance in the meta-analysis of the 1 df test for G × Vitamin interaction. While it remains quite difficult to obtain definitive evidence for G × E interaction in genome-wide studies, perhaps due to small effect sizes of individual genes combined with low exposure rates, this analysis of two large case–parent trio studies argues for considering possible G × E interaction in any comprehensive study of complex and heterogeneous disorders such as OFC.

## Introduction

Orofacial clefts (OFCs) are the most common craniofacial malformations in humans, affecting approximately one per 1,000 live births (Mai et al., 2014). OFCs are commonly categorized into two anatomically and embryologically distinct entities: cleft lip with or without cleft palate (CL/P) and cleft palate alone (CP) (Jiang et al., 2006). Among all infants born with an OFC, 70% of CL/P cases and 50% of CP cases occur as isolated, non-syndromic malformations (Shi et al., 2008). Non-syndromic CL/P occurs more frequently in males than females (2:1) whereas non-syndromic CP occurs more often in females (Mossey et al., 2009). Substantial variation in birth prevalence rates of non-syndromic CL/P has been reported across populations: Asian populations have higher birth prevalence rates compared to those of European descent (Dixon et al., 2011) and African populations have the lowest birth prevalence rates (Mossey et al., 2009). Compared to CL/P, non-syndromic CP shows less variability in birth prevalence rates across populations (Genisca et al., 2009; Beaty et al., 2011). Due to the high overall birth prevalence rate and the large financial, medical, and emotional burden of treatment required by children with an OFC, understanding the etiology of OFCs is an important public health goal.

Genome-wide association studies (GWAS) using both case–control (Birnbaum et al., 2009; Mangold et al., 2010) and case–parent trio designs (Beaty et al., 2010, 2011, 2013; Leslie et al., 2016a) have identified multiple genetic risk factors for OFCs. There have been multiple GWAS for CL/P (Birnbaum et al., 2009; Grant et al., 2009; Beaty et al., 2010; Mangold et al., 2010; Camargo et al., 2012; Sun et al., 2015; Wolf et al., 2015; Leslie et al., 2016a; Yu et al., 2017; Butali et al., 2019; Huang et al., 2019), two genome-wide meta-analysis of CL/P (Ludwig et al., 2012; Leslie et al., 2017), and four GWAS of CP (Beaty et al., 2011; Leslie et al., 2016b; Butali et al., 2019; He et al., 2020). These studies have revealed a complex genetic architecture controlling risk to OFCs. More than 40 different genes or regions have yielded genome-wide significant associations with risk to CL/P from multiple populations, while one gene (*GRHL3*) has been clearly identified as associated with risk to CP [largely limited to populations of European ancestry (Leslie et al., 2016b)]. A recent case–control study of Han Chinese CP cases and controls also identified the region on chromosome 15q24.3 as associated with risk of CP (He et al., 2020). Of these recognized risk genes achieving genome-wide significance, four regions (*IRF6* on 1q32-41, the gene desert on 8q24, markers on 10q25.3 and on 17q22) can explain about a quarter of the estimated heritability in risk to CL/P based on twin and family studies (Beaty et al., 2016; Lupo et al., 2019), which has been estimated to be around 90% for both CL/P and CP based on twin registry data in European populations (Grosen et al., 2011). Thus, additional genetic risk factors likely remain to be identified.

In addition to a strong genetic component to risk for OFCs, several environmental risk factors contribute to its etiology. For example, maternal smoking (Honein et al., 2014), passive exposure to cigarette smoke (Kummet et al., 2016), and binge alcohol consumption (Romitti et al., 2007) have been reported to significantly increase risk of OFCs, while multivitamin supplementation appears to play a protective role (Johnson and Little, 2008). Whenever there is some effect of an environmental risk factor, it is important to test for potential gene–environment (G × E) interaction, where the joint risk of exposure and a genetic risk factor may become more important than predicted by the respective marginal effects of the gene or the exposure. While it is quite difficult to prove the existence of G × E interaction based on statistical evidence alone (Aschard, 2016), there are some examples of possible G × E interactions relevant to OFCs. For example, variants in the *GRID2* and *ELAVL2* genes showed evidence of G × E interaction with maternal smoking in influencing the risk of CL/P among mothers of European ancestry (Beaty et al., 2013). A Brazilian sample of case–parent trios yielded suggestive evidence for G × E interaction between a marker in *RAD51*, a DNA repair gene, and risk of CL/P (Machado et al., 2016). Moreover, variants in *SMC2* on chromosome 9 appeared to increase the risk of CP in the presence of maternal drinking, while variants in *BAALC* on chromosome 8 appeared to reduce risk of CP in the presence of multivitamin supplementation (Beaty et al., 2011). Although it has been widely acknowledged OFCs result from a complex interplay of genetic and environmental risk factors, specific evidence for G × E interaction remains tentative at best.

In this paper, we used a trio-based design to explore possible G × E interaction effects using two large multi-ethnic studies of case–parent trios: the Gene, Environment Association (GENEVA) consortium and case–parent trios drawn from the Pittsburgh Orofacial Cleft (POFC) study. Both studies have genome-wide marker data available and additional markers were imputed against the same reference panel (1000G phase 3 v5). The case–parent trio design provides a unique advantage when analyzing samples from distinct populations for a relatively rare disorder. Unlike a cohort study with randomly ascertained individuals or the more conventional case–control study design, the case–parent trio design is robust to spurious signals arising from population stratification (a form of confounding due to differences in marker allele frequencies and disease risk across genetically distinct sub-populations), which can occur whenever samples from multiple populations are combined. We used the genotypic transmission disequilibrium test (gTDT) to test for possible G × E interactions considering three common maternal exposures (maternal smoking, maternal alcohol consumption, and maternal vitamin supplementation in the 3 months before conception through the first trimester).

## Materials and Methods

### GENEVA Study Samples

The samples in the GENEVA consortium include case–parent trios from multiple populations combined in a GWAS of non-syndromic OFC. Case–parent trios were recruited largely through surgical treatment centers by multiple investigators from Europe (Norway), the United States (Iowa, Maryland, Pennsylvania, and Utah) and Asia (People’s Republic of China, Taiwan, South Korea, Singapore, and the Philippines) (Beaty et al., 2010, 2011; Leslie et al., 2017). Phenotypes (e.g., type of cleft), sex, race, as well as common environmental risk factors [e.g., maternal smoking, environmental tobacco smoke (ETS), multivitamin supplementation, and alcohol consumption during the periconceptual period] were obtained through direct maternal interview (Beaty et al., 2010, 2011). The research protocol was approved by the Institutional Review Boards (IRBs) at the Johns Hopkins Bloomberg School of Public Health and at each participating recruitment site. Written informed consent was obtained from both parents, and assent from the case was solicited whenever the child was old enough to understand the purpose of the study. Originally, 412 individuals from POFC were included in GENEVA (Leslie et al., 2016a) and these duplicated samples were subsequently removed from the GENEVA samples used here, so these GENEVA and POFC trios represent independent, non-overlapping case–parent trios from three major racial/ethnic groups (European, Asian, and Latin American).

### POFC Study Samples

The POFC study included case–parent trios ascertained through a proband with an isolated CL/P or CP from multiple populations and a large number of OFC cases and ethnically matched controls from some of these same populations (Leslie et al., 2016a, 2017). However, in this analysis, only unrelated case–parent trios from POFC were used. The distribution of trios by cleft subtype (CL/P and CP) and racial/ethnic groups from both studies is given in Table 1.

**TABLE 1:** Number of case–parent trios in the GENEVA and the POFC studies stratified by type of cleft (CL/P and CP) and racial/ethnic group (European, Asian, and Latin American).

			**All CL/P**	**Euro. CL/P**	**Asian CL/P**	**All CP**	**Euro. CP**	**Asian CP**	**Latin Am. CL/P**	**Latin Am. CP**
**GENEVA Study**										
Trios before individual filtering ^[1]^			1591	668	895	466	215	237		
Trios after individual filtering ^[2]^			1487	575	891	452	203	235		
Exposure	Environ. tobacco smoke	Trios ^[3]^	1254	454	784	403	158	232		
		Exposed trios	370 (30%)	64 (14%)	300 (38%)	116 (29%)	22 (14%)	94 (41%)		
	Maternal Smoking	Trios ^[3]^	1485	573	891	452	203	235		
		Exposed trios	208 (14%)	179 (31%)	26 (3%)	65 (14%)	57 (28%)	7 (3%)		
	Multivitamin	Trios ^[3]^	1258	486	752	397	180	205		
		Exposed trios	430 (34%)	287 (59%)	131 (17%)	170 (43%)	111 (62%)	49 (24%)		
	Alcohol	Trios ^[3]^	1474	573	880	449	202	233		
		Exposed trios	249 (17%)	227 (40%)	19 (2%)	94 (21%)	83 (41%)	9 (4%)		
**POFC Study**										
Trios before individual filtering ^[1]^			1319	406	284	165	93	38	601	29
Trios after individual filtering ^[2]^			1284	403	284	159	93	38	597	28
Exposure	Environ. tobacco smoke	Trios								
		Exposed trios								
	Maternal Smoking	Trios ^[3]^	953	339	127	120	81	13	487	26
		Exposed trios	155 (16%)	70 (21%)	8 (6%)	18 (15%)	15 (19%)	1 (8%)	77 (16%)	2 (8%)
	Multivitamin	Trios ^[3]^	770	249	127	94	74	14	394	6
		Exposed trios	565 (73%)	208 (84%)	100 (79%)	71 (76%)	55 (74%)	13 (93%)	257 (65%)	3 (50%)
	Alcohol	Trio ^[3]^	860	249	127	115	76	13	484	26
		Exposed trios	195 (23%)	91 (37%)	10 (8%)	29 (25%)	18 (24%)	2 (15%)	94 (19%)	9 (35%)

*^[1]^Number of complete trios in the original GENEVA/POFC dataset. ^[2]^Number of complete trios after quality control. ^[3]^Number of complete trios after excluding those with missing information on maternal exposure.*

Similar to the GENEVA study, the three environmental risk factors (e.g., maternal smoking, multivitamin supplementation, and alcohol consumption during the 3 months before conception and for each trimester of pregnancy) were obtained through direct maternal interview. Exposure to ETS, however, was not available in the POFC data. The research protocol was approved by the IRBs at the University of Pittsburgh and all participating institutions, and informed consent was obtained from all participants.

### Genotyping and Imputation

In the GENEVA study, DNA was genotyped at the Center for Inherited Disease Research^1^ on the Illumina Human610 Quadv1_B array, which includes 589,945 SNPs through the NHGRI GENEVA program and submitted to dbGaP (^2^ accession number phs000094.v2.p1). To take advantage of more efficient imputation tools and larger reference panels, we re-imputed genotypes on the GENEVA dataset using the Michigan Imputation Server (Das et al., 2016) after dropping very low frequency SNPs (i.e., those with minor allele frequency or MAF < 0.01) and phasing haplotypes from the observed genotypes using SHAPEIT (Delaneau et al., 2011) while considering family structure (Taub et al., 2012). This imputation tool provides an efficient computation with comparable accuracy to traditional imputation tools (e.g., IMPUTE2). The reference panel was “1000G phase 3 v5” as used on POFC data. For quality control purposes, all genotyped SNPs with missingness > 5%, Mendelian error rate > 5%, those having a MAF < 5%, as well as SNPs showing deviation from Hardy-Weinberg equilibrium (HWE) at *p* < 10^–4^ among parents were dropped, following the procedures used with POFC (Carlson et al., 2017; Leslie et al., 2017). All imputed SNPs were filtered to exclude any with an *R*^2^ < 0.3 with BCFtools-v1.9^3^. Additionally, individuals with low-quality DNA, individuals with SNP missingness > 10%, and individuals duplicated across the POFC and GENEVA datasets were removed. Only complete trios were kept for the final analysis. The final GENEVA dataset contained 6,762,077 SNPs (including both observed and imputed SNPs with MAF > 5% among parents) for 1,939 complete case–parent trios (including 1,126 Asian and 778 European trios).

The case–parent trios from the POFC study were genotyped for 539,473 SNPs using the Illumina HumanCore + Exome array (available through dbGAP accession number phs000774.v2.p1), and similar quality control filtering was used to remove rare and poor-quality SNPs. Genomic coordinates were given in human genome build 37 (hg19). Genotype data were pre-phased with SHAPEIT taking family structure into account (Taub et al., 2012) and then imputed with IMPUTE2 using the 1000 Genomes Phase 3 reference panel as described previously (Leslie et al., 2016a). Incomplete trios, trios with parents from different racial/ethnic groups and ethnic groups with insufficient sample sizes for effective imputation were dropped from the analyses. The final POFC trio dataset analyzed here contained 6,350,243 SNPs (including both observed and imputed SNPs with MAF > 5% among parents) for 1,443 complete case–parent trios (including 322 Asian, 625 Latin American, and 496 European trios).

### Statistical Analysis

Because larger sample sizes are required to detect G × E interaction effects compared to the marginal effects of genes alone (Aschard, 2016), here we deliberately combined case–parent trios from all recruitment sites within each study and pooled both of the major subgroups of OFC (i.e., CL/P and CP) to maximize sample size. Our goal in pooling is not to identify G × E effects specific to one cleft subgroup but to identify G × E effects present in one or both subgroups. Thus, findings of G × E effects in our “all OFC” group should be interpreted as such. It is worth noting that pooling CL/P and CP trios increases the chance of missing signals when true interaction effects exist only in one cleft subgroup (i.e., increased false negatives or reduced power) but does *not* result in spurious findings (i.e., unchanged false positives or controlled type I error). However, reduced power due to genetic heterogeneity is counter-balanced by improved power due to increased sample size when genetic sharing between CL/P and CP exists (Leslie et al., 2017; Carlson et al., 2019; Ray et al., 2020).

For our G × E interaction analyses, we considered three self-reported maternal exposures: maternal smoking, alcohol consumption, and multivitamin supplementation. Note that ETS was not available in both GENEVA and POFC, and hence not studied in this analysis. We used the gTDT in the R *trio* package (Schwender et al., 2014) for this case–parent trio study to test the null hypothesis of independence between each common SNP and no interaction with these environmental risk factors. Closed-form solutions were used to estimate the regression coefficients and their respective standard errors under a conditional logistic regression model for different genetic models (recessive, dominant, or additive) while allowing efficient implementation on a genome-wide scale (Schwender et al., 2012). The *trio* package (v3.20.0^4^) was used on common SNPs in the combined set of all OFC trios from GENEVA and POFC separately. For a common bi-allelic marker, a conditional logistic model can be used to test the null hypothesis of independence between each common SNP and disease (or equivalently, the composite null hypothesis of no linkage *or* no association between a SNP and an unobserved causal variant). In this article, we assume an additive genetic model and consider the conditional logistic model that models the association between each common SNP and its interaction with a maternal exposure and OFC:

$$P\left(Y_{0}=1|\left\{\sum_{l=0}^{3}Y_{l}=1\right\},\left\{G_{0},G_{1},G_{2},G_{3}\right\},E\right)=\frac{e^{\beta_{G}*G_{0}+\beta_{G\,E}*G_{0}*E}}{\sum_{l=0}^{3}e^{\beta_{G}*G_{l}+\beta_{G\,E}*G_{l}*E}}$$

where *Y_0* is the disease status of the child (taking the value 1 for the observed child in a case–parent trio study); *Y_l* is the disease status of the *l*th pseudo-control (taking value 0 for all pseudo-controls, *l*=1,2,3); *G_0* is the genotype of the child (case) at the marker coded additively as 0, 1, or 2; *G_l* is the genotype of the *l*th pseudo-control at this same marker; and *E* is a binary environmental variable denoting presence/absence of a maternal exposure during pregnancy. Basically, *G_1*, *G*_2_, and *G_3* represent possible SNP genotypes the observed case did not inherit from the parents. We first performed a 2 df χ^2^ test of the null hypothesis *H*_0_:β_*G*_=0,β_*GE*_=0 to identify markers with either a main effect, or a G × E interaction effect, or both. To focus exclusively on G × E interaction between a marker and a maternal exposure, we conducted a 1 df χ^2^ test of the null hypothesis $H_{0}^{\left(G\,E\right)}:\beta_{G\,E}=0$ within each dataset.

Finally, we conducted a combined analysis of the GENEVA and the POFC studies using inverse-variance weighted fixed effect meta-analysis. The closed form solutions of the coefficients and their standard errors from the gTDT model discussed above enable computationally efficient genome-wide meta-analysis across both studies. In particular, for the 1 df G × E interaction test, if ${\hat{\beta }}_{G\,E,1}$ and ${\hat{\beta }}_{G\,E,2}$ represent the G × E coefficient estimates from the two studies, and ${\hat{S\,E}}_{G\,E,1}$ and ${\hat{S\,E}}_{G\,E,2}$ are their respective estimated standard errors (all of which are output from the *trio* package), then the overall meta-analyzed estimates are ${\hat{\beta }}_{G\,E}=\frac{\sum_{i=1,2}{\hat{\beta }}_{G\,E,i}\,\omega_{i}}{\sum_{i=1,2}\omega_{i}}$ and ${\hat{S\,E}}_{G\,E}=\sqrt{\frac{1}{\sum_{i=1,2}\omega_{i}}}$, where $\omega_{i}=\frac{1}{{\hat{S\,E}}_{G\,E,i}^{2}}$ for *i*=1,2. We calculated these meta-analyzed estimates ${\hat{\beta }}_{G\,E}$ and ${\hat{S\,E}}_{G\,E}$ using the R package *meta* (v4.13.0) (Balduzzi et al., 2019) and applied a 1 df χ^2^ test of the null hypothesis $H_{0}^{\left(G\,E\right)}:\beta_{G\,E}=0$ for each marker and three maternal exposures (smoking, alcohol consumption, and multivitamin supplementation). To account for multiple comparisons in this genome-wide analysis, we used the conventional threshold of 5 × 10^–8^ to declare genome-wide significance but also investigated SNPs yielding only suggestive evidence of G × E interaction effects (*p* < 10^–6^). For the 2 df G × E interaction test, we meta-analyzed using the approach described in Manning et al. (2011). Specifically, we implemented the 2 df χ^2^ test of *H*_0_:β_*G*_=0,β_*GE*_=0 by jointly meta-analyzing estimates ${\hat{\beta }}_{G,1}$, ${\hat{\beta }}_{G\,E,1}$, ${\hat{\beta }}_{G,2}$, ${\hat{\beta }}_{G\,E,2}$, ${\hat{S\,E}}_{G,1}$, ${\hat{S\,E}}_{G\,E,1}$, ${\hat{S\,E}}_{G,2},$ and ${\hat{S\,E}}_{G\,E,2}$ across these two studies using 6,761,961 SNPs including those present in both datasets and those unique to one dataset if the allele calls and position were consistent. Our R code for this 2 df joint meta-analysis of main and interaction effects can be found at https://github.com/RayDebashree/GxE.

Manhattan plots and QQ plots were created for each analysis to show signals and to check for potential bias in the test statistic, respectively (Taub et al., 2012). The genomic inflation factors (λ) were calculated using the “estlambda” function with the “median” option from the GenABEL R package v1.8-0 (Aulchenko et al., 2007). SNPs achieving significance from the gTDT analyses were annotated with an online tool SNPnexus^5^ (Dayem Ullah et al., 2012) to identify potentially important genes. Regional association plots generated using LocusZoom^6^ (Pruim et al., 2010) were used to examine detailed evidence of association for each region achieving or approaching genome-wide significance under an additive model in the combined meta-analysis. For these LocusZoom plots, we used genome build hg19 with no specified linkage disequilibrium (LD) reference panel due to the multi-ethnic nature of these two datasets.

## Results

### Meta-Analysis of G and G × E Interaction Effects in the 2 df Test

**FIGURE 1:**
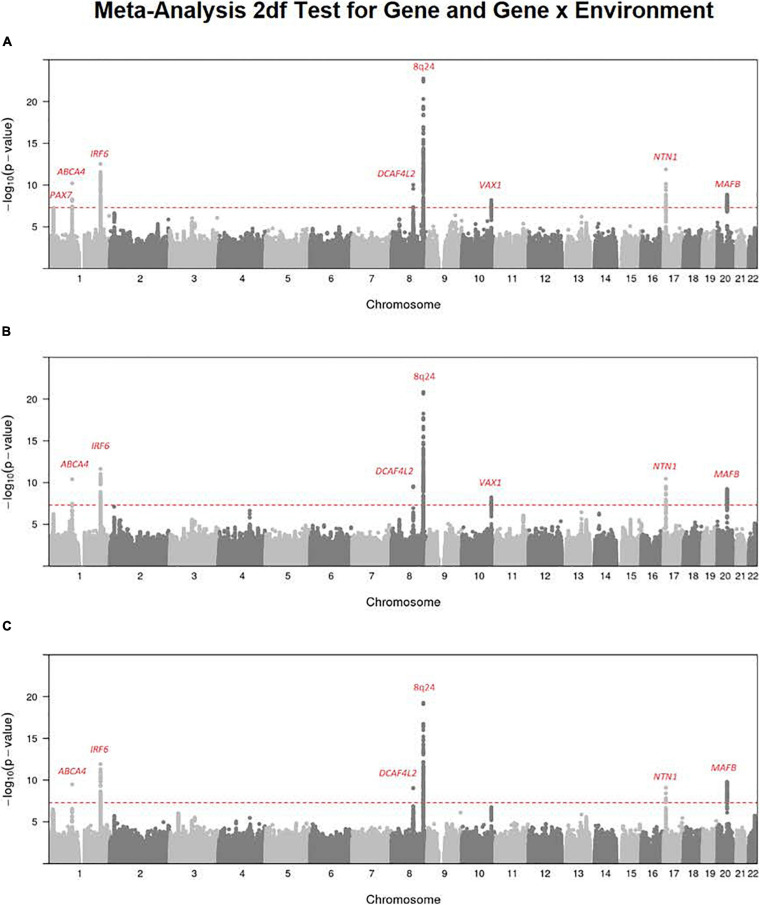
Meta-analysis of the 2 df joint test for G and G × E interaction on all OFC case–parent trios across both the GENEVA and POFC studies. Numerous genes/regions show strong evidence of influencing risk to OFC largely through the main gene effect (β_*G*_) with very subtle differences that could be attributed to G × E interaction effect (β_*GE*_). Most of these strong signals represent recognized risk genes for CL/P. **(A)** Meta-analysis of 2 df test for G and G × Smoking interaction. **(B)** Meta-analysis of the 2 df test for G and G × Alcohol interaction. **(C)** Meta-analysis of the 2 df test for G and G × Vitamin interaction. The red dashed line represents the conventional threshold for genome-wide significance (5 × 10^–8^).

It has been suggested the 2 df joint test for gene (G) and G × E interaction could provide more power to detect genes influencing risk to complex and heterogeneous diseases when there is any possibility of G × E interaction (Kraft et al., 2007). Figure 1 shows the Manhattan plot from a meta-analysis across both studies of this joint 2 df test for all three available exposures for all OFC case–parent trios (corresponding QQ plots for this 2 df test are shown in Supplementary Figure 1). Clearly, the multiple recognized risk genes/regions yielding strong evidence of linkage and association for CL/P dominate the statistical results shown in Figure 1. These different peaks represent recognized risk genes for CL/P (e.g., *PAX7* on 1p36, *ABCA4* on 1p22, *IRF6* on 1q32, *DCAF4L2* on 8q21, the 8q24 gene desert region, *VAX1* on 10q25.3, *NTN1* on 17p13.1, and *MAFB* on 20q12). This meta-analysis does show recognized risk genes for OFCs are not obscured in this 2 df joint test of G and G × E interaction.

**FIGURE 2:**
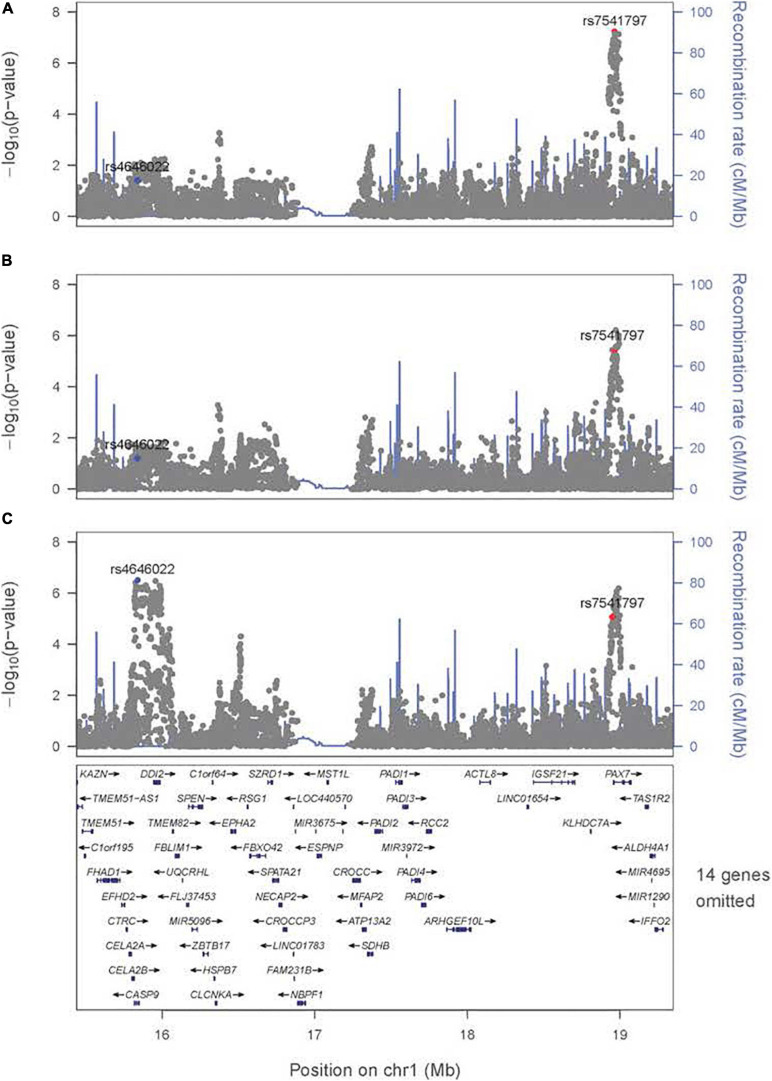
Significance of the 2 df test considering G and G × E interaction for the region on 1p36 encompassing *CASP9* and *PAX7*. Regional association plot for the 2 df test for **(A)** G and G × Smoking interaction, **(B)** G and G × Alcohol interaction, and **(C)** G and G × Vitamin interaction. The most significant SNP (rs4646022) in the 1 df test for G × Vitamin interaction is denoted in blue; the most significant SNP (rs7541797) in the 2 df test for G and G × Smoking interaction is denoted in red.

There are some differences among these results from meta-analysis across the three exposures considered (i.e., across panels A–C in Figure 1), and their differences must arise from the estimated G × E interaction parameter (β_*GE*_). For example, the signal for SNPs near *PAX7* on 1p36 almost achieved genome-wide significance for the joint test of G and G × Smoking (Figure 1A) where the top SNP (rs7541797) gave *p* = 5.5 × 10^–8^ in the 2 df test, but was less significant when G and G × Alcohol (*p* = 3.4 × 10^–6^) and when G and G × Vitamin (*p* = 8.6 × 10^–6^) were analyzed in this joint test (Figures 1B,C). In fact, this peak on 1p36 was joined by a second peak 3.2 Mb telomeric of *PAX7* that encompassed *CASP9* in the 2 df test for G and G × Vitamin interaction, sufficient physical distance to result in very weak LD between top SNPs in these two genes (all *r*^2^ < 0.1). Specifically, SNP rs4646022 yielded suggestive significance for G and G × Vitamin interaction in this 2 df test (*p* = 3.1 × 10^–7^). Figure 2 shows the region of 1p36 encompassing *CASP9* and *PAX7* for the 2 df joint test of G and G × E interaction for each of the three maternal exposures. Figures 2A,B show a clear peak near *PAX7* and virtually no signal in the region of *CASP9* (n.b. the peak SNP from the 2 df test for G and G × Smoking interaction is noted by the red dot, while the blue dot represents SNP rs4646022). Figure 2C where G and G × Vitamin interaction was considered, however, shows considerable support against the null hypothesis for both genes although multiple genes are located within this region around *CASP9*.

### Meta-Analysis of the 1 df Test for Maternal Smoking Interaction

**FIGURE 3:**
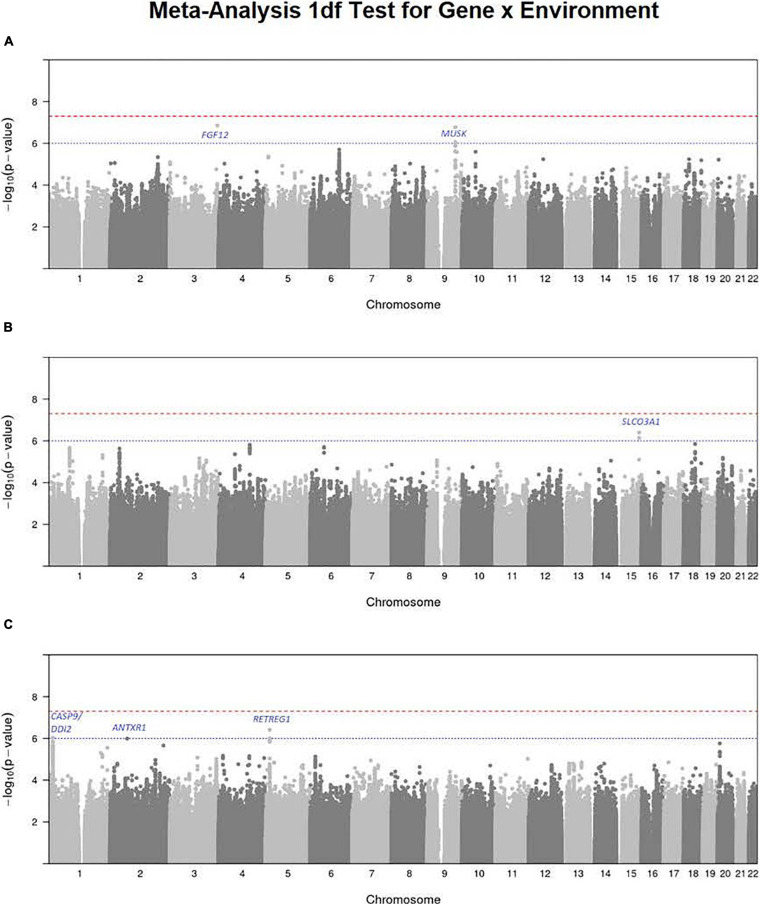
Manhattan plots for the 1 df test for G × E interaction from meta-analysis over both GENEVA and POFC studies for **(A)** maternal smoking, **(B)** maternal alcohol consumption, and **(C)** maternal multivitamin supplementation. The red dashed line represents conventional critical value for genome-wide significance (5 × 10^–8^) and the blue dotted line represents a less stringent threshold (10^–6^) for “suggestive” evidence of G × E interaction.

**FIGURE 4:**
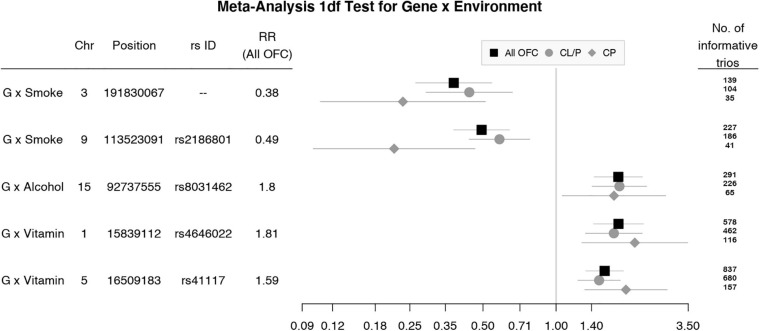
Estimated relative risks (RR) and 95% CI for SNPs listed in Table 2 from the 1 df test of G × E interaction in meta-analysis of all OFC trios (black squares) and stratified analysis of CL/P (gray circles) and CP trios (gray diamonds).

To focus explicitly on tests of G × E interaction, we used the 1 df test for H_0_:β_*GE*_ = 0 over all SNPs (observed and imputed) in a similar meta-analysis over both the GENEVA and the POFC studies (Figure 3A with the corresponding QQ plot in Supplementary Figure 2A). While no SNPs achieved formal genome-wide significance in this meta-analysis, several genes did yield suggestive evidence (with *p* < 10^–6^) of possible G × Smoking interaction and may warrant further exploration. Table 2 lists the most significant SNPs (and their nearest genes) for each region showing suggestive evidence in the meta-analysis, noting which allele was the effect allele, along with its corresponding estimated relative risk (RR) of G × E interaction, 95% confidence interval (CI), *p* value, and frequency in each racial/ethnic group. Figure 4 shows the RR estimates and their 95% CI for each of these top SNPs from the meta-analysis and from stratified analyses based on CL/P and CP groups separately. There is consistency in the estimated effect sizes and directions within each stratum, and as expected, the 95% CIs are always larger for the CP group due to their smaller sample size.

**TABLE 2:** Markers exceeding the threshold for “suggestive” evidence (*p* < 10^–6^) in the 1 df test for G × E interaction from meta-analysis over GENEVA and POFC case–parent trios for all OFC.

**Chr**	**Position**	**rs ID**	**Nearest Gene**	**Relative Risk [95% CI]**	***p* value**	**Effect Allele Frequency**		
						**Euro.**	**Asian**	**Latin Am.**
**G × Smoking interaction 1 df test in meta-analysis**								
3	191830067	–	*FGF12*	0.379 [0.264, 0.544]	1.37 × 10^–7^	0.24	0.05	–
9	113523091	rs2186801: C:G^∧^	*MUSK*	0.494 [0.379, 0.643]	1.68 × 10^–7^	0.20	0.33	0.41
**G × Alcohol interaction 1 df test in meta-analysis**								
15	92737555	rs8031462: T:C^∧^	*SLCO3A1*	1.804 [1.436, 2.267]	3.99 × 10^–7^	0.48	0.17	0.42
**G × Vitamin interaction 1 df test in meta-analysis**								
1	15839112	rs4646022: G:A^∧^	*CASP9*	1.807 [1.427, 2.288]	9.06 × 10^–7^	0.23	0.08	0.18
5	16509183	rs41117: A:G^∧^	*RETREG1*	1.585 [1.327, 1.894]	3.87 × 10^–7^	0.39	0.63	0.41

*^∧^Effect allele.*

**FIGURE 5:**
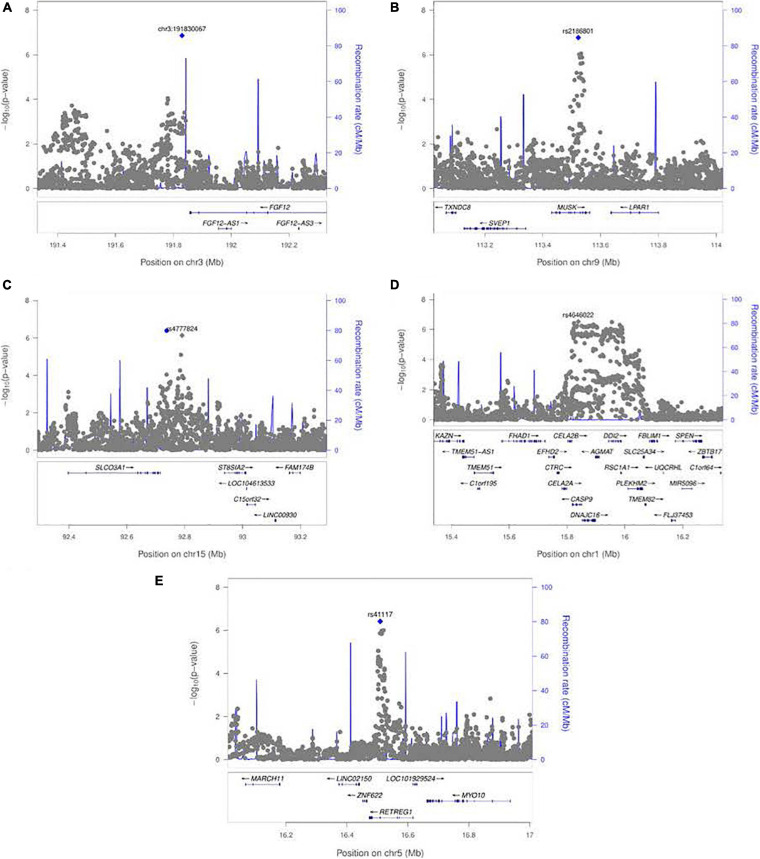
LocusZoom plots for the 1 df test of G × E interaction between maternal exposures and SNPs near their respective peak signals from meta-analysis over both GENEVA and POFC studies. **(A)** Peak on chr. 3 near *FGF12* from the test for G × Smoking interaction shown in Figure 3A. **(B)** Peak on chr. 9 near *MUSK* from the test for G × Smoking interaction shown in Figure 3A. **(C)** Peak on chr. 15 near *SLCO3A1* from the test for G × Alcohol interaction shown in Figure 3B. **(D)** Peak on chr. 1 near *CASP9* for G × Vitamin interaction shown in Figure 3C. **(E)** Peak on chr. 5 near *RETREG1* from the test for G × Vitamin interaction shown in Figure 3C.

A polymorphic insertion/deletion (indel) at position 191,830,067 on 3q28-q29 near *FGF12* and an intronic SNP rs2186801 in the 9q31.3 region containing the *MUSK* (muscle associated receptor tyrosine kinase) gene both gave such suggestive evidence when testing for G × Smoking interaction. The top signal near *FGF12* is questionable, however, because nearby SNPs did not show any supporting evidence of linkage and association (see Figure 5A), and this polymorphic indel was only imputed in the GENEVA study with somewhat reduced quality (*R*^2^ = 0.84). As indels are intrinsically more difficult to call, extreme caution should be used when interpreting suggestive evidence of possible G × Smoking interaction. Also, the frequency of the allele associated with any effect on risk showed considerable variability across racial/ethnic groups (Table 2). The peak on 9q31.3 is, however, more interesting where several SNPs in and near *MUSK* gave suggestive evidence. Figure 5B shows greater resolution for this region where multiple SNPs yielded suggestive evidence of G × Smoking interaction, and the peak SNP (rs2186801) had *p* = 1.68 × 10^–7^, with the G allele having an apparent protective effect on risk (Table 2). This imputed SNP was highly polymorphic in all racial/ethnic groups in these two datasets.

### Meta-Analysis of the 1 df Test for Maternal Alcohol Consumption

Meta-analysis over the GENEVA and the POFC studies was conducted using the 1 df test for G × Alcohol interaction for all observed and imputed SNPs (Figure 3B with the corresponding QQ plot shown in Supplementary Figure 2B). Only 2 SNPs (imputed SNPs rs8031462 and rs4777824) near *SLCO3A1* on 15q26 achieved suggestive evidence of linkage and association (*p* = 4.0 × 10^–7^ and *p* = 7.2 × 10^–7^, respectively, with the former listed in Table 2). Figure 5C shows this peak region at greater resolution where multiple SNPs yielded nominal evidence of G × Alcohol interaction.

### Meta-Analysis of the 1 df Test for Maternal Vitamin Supplementation

Meta-analysis was conducted on all OFC case–parent trios using the 1 df test for G × Vitamin interaction (Figure 3C with corresponding QQ plot shown in Supplementary Figure 2C). As seen with the 2 df test discussed above, the suggestive peak seen on 1p36 reflected SNPs near the *CASP9* gene in the meta-analysis of this 1 df test (with the same SNP rs4646022 mentioned above yielding *p* = 9.06 × 10^–7^ in this 1 df test for G × Vitamin interaction). Figure 5D shows this evidence of linkage and association around rs4646022 in greater detail and reveals a broad region of statistical signal against the null hypothesis of no G × Vitamin interaction. While many genes fall in this region, the *CASP9* gene is of interest because it has been previously associated with risk of OFC (Holzinger et al., 2017).

Also, one imputed SNP (r68079474) in *ANTXR1* on 2p13.3 approached (but did not exceed) the threshold for “suggestive” significance (*p* = 1.02 × 10^–6^) in this 1 df test for G × Vitamin interaction (see Supplementary Figure 3). Caution must be used in interpreting this observation, however, because this variant had a low frequency in all racial/ethnic groups (0.08 among parents of European ancestry, 0.04 among parents of Asian ancestry, and 0.12 among Latin American parents).

Another imputed SNP rs41117 located near *RETREG1* (a.k.a. *FAM134B*) on 5p15.1 also achieved suggestive evidence for G × Vitamin interaction in this 1 df test. Figure 5E shows the suggestive evidence of linkage and association around rs41117 in greater detail and Table 2 lists its estimated effect sizes and allele frequencies in each of the major racial/ethnic groups. This imputed SNP was highly polymorphic in all groups.

## Discussion

While it is widely accepted that both genes and environmental risk factors influence risk to OFC, it is quite difficult to formally test for statistical interaction between the two (Aschard, 2016). Statistical interaction is defined as an observable deviation (either increasing or decreasing risk) from the predicted joint effect of a gene and an environmental risk factor based on their respective estimated marginal effects. Detecting G × E interaction requires larger sample sizes than necessary to estimate their respective main effects (perhaps more than is feasible to accumulate for low prevalence diseases), especially when the measure of exposure to the environmental risk factor is crude and imprecise, e.g., a simple binary classification of exposed vs. unexposed. Here, we have tried to maximize sample size by considering two large family-based studies of OFC each recruiting case–parent trios from multiple populations and pooling CL/P and CP into an all OFC group. Our findings of G × E effects in this manuscript should be interpreted as G × E signals that may be present in one or both cleft subgroups.

This strategy to maximize sample size by pooling together all OFC subtypes carries its own risks due to the documented genetic heterogeneity between CL/P and CP; chiefly multiple different genes have been shown to influence risk for CL/P, but there are fewer recognized genetic risk factors for CP. Historically, these two subgroups of OFC have been thought to have distinct etiologies based on developmental and epidemiologic patterns. Recently, some studies reported evidence of shared genetic risk for variants in *IRF6*, *GRHL3*, and *ARHGAP29* regions (Schutte et al., 1993; Beaty et al., 2010; Liu et al., 2017a,b). Leslie et al. (2017) found that variants near *FOXE1* influenced risk of both CL/P and CP in GWAS from both the GENEVA and the POFC studies but including additional case–control subjects from POFC. There is some evidence that markers can show association with risk to CL/P and CP in opposite directions. For instance, markers near *NOG* on 17q22 have shown weak evidence of decreased risk for one OFC subgroup and increased risk for the other (Moreno Uribe et al., 2017; Carlson et al., 2019). In a parallel study, our group has explored genetic overlap between OFC subtypes using a new statistical approach, PLACO (Ray and Chatterjee, 2020). In that study, we not only found loci in/near recognized OFC genes and some novel genes exerting shared risk but also identified some genetic regions with apparently opposite effects on risk to CL/P and CP (Ray et al., 2020).

Furthermore, when we pool samples from multiple racial/ethnic groups, different populations will vary in the statistical support for different genes (due to variation in allele frequencies and underlying patterns of LD), but also just due to different exposure rates. If sub-populations differ in both their allele frequencies and their exposure rates (i.e., if the two are correlated in the combined sample), an estimation approach can be used to estimate mean exposure rates within distinct sub-populations, which may protect from spurious results in tests for G × E interaction (Shin et al., 2012). However, if the overall exposure rate is simply too low in one or more sub-populations, there will be little statistical power to estimate or test for G × E interaction, and the results from any analysis of combined samples will be dominated by those sub-populations with higher exposure rates. It would be difficult to predict specific circumstances under which countervailing effects on risk could enhance or negate evidence for marginal effects of individual genetic risk factors and their potential G × E interaction effects.

The 2 df test from the meta-analysis of all OFC case–parent trios revealed many recognized risk genes for CL/P, the predominant form of OFC in this study. Confirmed risk genes include the following: *PAX7* (1p36.13), *ABCA4* (1q22), *IRF6* (1q32.2), *DCAF4L2* (8q21.3), 8q.24 (gene desert), *VAX1* (10q25.2), *NTN1* (17p13.1), and *MAFB* (20q12). This is reassuring and argues that testing for possible G × E interaction will not conceal genetic risk factors when they do exist. Also, G × E interaction may not be an overwhelming risk factor for OFC controlled by these well-recognized risk genes.

The region on 1p36.13 includes the well-recognized risk gene *PAX7* but interestingly when G × Vitamin interaction was included in the conditional logistic model for the gTDT, a rather distinct suggestive peak becomes apparent a short physical distance from *PAX7* (see Figure 2C). While this peak encompasses many genes, *CASP9* (caspase 9) is of particular interest because it was previously identified as a potential risk gene for OFC based on a sequencing study of members of multiplex cleft families from Syria (Holzinger et al., 2017). In this study, a rare, non-synonymous variant in *CASP9* (predicted to be pathogenic) occurred in three homozygous family members with an OFC as well as other affected relatives who were heterozygous. *CASP9* is directly involved in an apoptotic signaling pathway shown to result in a facial cleft phenotype in mouse models (D’Amelio et al., 2010). A more recent sequencing study of Chinese cases with a neural tube defect (NTD) found more rare harmful variants in *CASP9* compared to matched controls and documented lower expression of this gene in cell culture when exposed to low folate levels (Liu et al., 2018). A recent whole exome sequencing study of two multiplex families with folate-resistant NTD showed variants in the intrinsic apoptotic pathway genes, *CASP9* and *APAF1*. These rare variants were loss-of-function changes occurring as compound heterozygous (Spellicy et al., 2018) and were approximately 1 Kb away from the rare variant reported in the multiplex cleft family (Holzinger et al., 2017). While both NTDs and OFCs are considered “mid-line birth defects” and studies have shown supplementation with folate and multivitamins can reduce risk to both (Wilson et al., 2015), it remains unknown if the same genes influence risk to both perhaps through G × E interaction.

When we focused on the 1 df test for evidence of G × E interaction alone, no markers achieved genome-wide significance, but several gave “suggestive” evidence and some of these are worthy of further consideration. Several SNPs in and near *MUSK* (muscle associated receptor tyrosine kinase) on 9p31.3 showed well-defined evidence against the null hypothesis (Figure 5B). The highly polymorphic SNP rs2186801 gave *p* = 1.68 × 10^–7^ with its G allele having an apparent protective effect on risk (Table 2). Mutations in *MUSK* are responsible for an autosomal recessive form of congenital myasthenic syndrome and a recessive form of fetal akinesia deformation sequence (FADS), providing support for its involvement in fetal development.

Two imputed SNPs (rs8031462 and rs4777824) near *SLCO3A1* (solute carrier organic anion transporter family member 3A1) on 15q26 yielded suggestive evidence G × Alcohol interaction (Figure 5C). The solute-carrier gene (SLC) superfamily encodes membrane-bound transporters and includes 55 gene families having at least 362 putatively functional protein-coding genes (He et al., 2009). These genes play an important role in transporting inorganic cations/anions (as well as vitamins) in and out of cells. There is suggestive evidence that *SLCO3A1* may be associated with nicotine dependence (Wang et al., 2012) and blood pressure through interaction with smoking (Montasser et al., 2009).

*RETREG1* (reticulophagy regulator 1; a.k.a. *FAM134B*) on 5p15.1 is a *cis-*Golgi transmembrane protein, and mutations in this gene lead to the production of an impaired gene product, which is unable to act as an autophagy receptor and leads to hereditary sensory and autonomic neuropathy in humans (HSAN IIB; OMIM 613135). This gene can also act as a tumor suppressor in colorectal adenocarcinoma and an oncogene in esophageal squamous cell carcinoma, and loss-of-function mutations can control viral replication (Islam et al., 2018).

This meta-analysis illustrates some of the strengths and challenges of searching for evidence of G × E interaction for complex and heterogeneous disorders such as OFC. Large sample sizes are needed, which inevitably result in both genetic heterogeneity and variation in exposure frequencies across subgroups. While combining the two anatomical forms of OFCs (CL/P and CP) together is unusual, it is reassuring that the estimated effect sizes in Figure 4 were always quite similar and showed the same direction of effect for those markers giving suggestive evidence of G × E interaction, although of course the 95% CIs were larger for the smaller CP group compared to the larger CL/P group. Ideally, we would like to have precise biomarkers of exposure (e.g., maternal cotinine measured during early pregnancy) rather than crude self-reported “yes/no” measures. In future studies, epigenetic markers may prove useful to confirm exposure, but validated epigenetic markers for early *in utero* exposures are not currently available. Even in large samples such as the two used here, statistical power may be limited, and statistical evidence may not achieve conventional genome-wide thresholds. Still in this manuscript, we have presented suggestive findings that may warrant further investigation to fully understand the etiology of OFC.

## Data Availability Statement

The datasets analyzed for this study can be found in dbGaP at (www.ncbi.nlm.nih.gov/gap) through dbGaP accession number phs000094.vl.pl (GENEVA) and accession number phs000774.v2.p1 (POFC), respectively.

## Ethics Statement

The studies involving human participants were reviewed and approved by the Institutional Review Boards (IRB) of each participating site, both domestic and foreign. For the GENEVA study, IRB at the Johns Hopkins Bloomberg School of Public Health and at each participating recruitment site aooroved. The research protocol for the POFC was approved by the IRB at the University of Pittsburgh and all participating institutions. Informed consent was obtained from all participants. Written informed consent to participate in this study was provided by the participants’ legal guardian/next of kin for minor children.

## Author Contributions

WZ did the re-imputation of the GENEVA genome-wide markers and conducted the statistical analyses of this dataset. SV ran the analyses of POFC data and the meta-analysis, summarized the findings, and generated the visualizations. JH managed the GENEVA dataset and directed quality control after imputation. MM, EF, SMW, and EL conducted data acquisition and analysis for the POFC. TB, JH, AS, IR, and MT conducted data acquisition and analysis for GENEVA. DR, MT, and IR designed and executed the statistical analysis. TB, DR, and WZ wrote the manuscript with input from JH, SV, MT, MM, IR, and AS. All authors contributed to the article and approved the submitted version.

## Conflict of Interest

The authors declare that the research was conducted in the absence of any commercial or financial relationships that could be construed as a potential conflict of interest.

## Footnotes

1 CIDR.jhmi.edu

2 www.ncbi.nlm.nih.gov/gap

3 https://samtools.github.io/bcftools

4 https://bioconductor.org/packages/release/bioc/html/trio.html

5 https://snp-nexus.org

6 http://locuszoom.org/
