# Supplementary material for: Detecting Gene-Environment Interaction for Maternal Exposures Using Case-Parent Trios Ascertained Through a Case With Non-Syndromic Orofacial Cleft
Source: Front Cell Dev Biol. 2021 Apr 16;9:621018. doi: 10.3389/fcell.2021.621018 (PMC8085423; doi:10.3389/fcell.2021.621018)

# Supplemental Material for "Detecting gene-environment interaction for maternal exposures using case-parent trios ascertained through a case with non-syndromic orofacial cleft" by Zhang et al. (2021)

**Figure S1**: QQ plots along with genomic inflation factors ($\lambda$) for the 2 df joint test for G and GxE interaction across three maternal exposures from meta-analysis of GENEVA and POFC shown in Figure 1. (A) QQ plot for 2 df test for joint effects of G and GxSmoking interaction; (B) QQ plot for 2 df test for joint effects of G and GxAlcohol interaction; (C) QQ plot for 2 df test for joint effects of G and GxVitamin interaction.

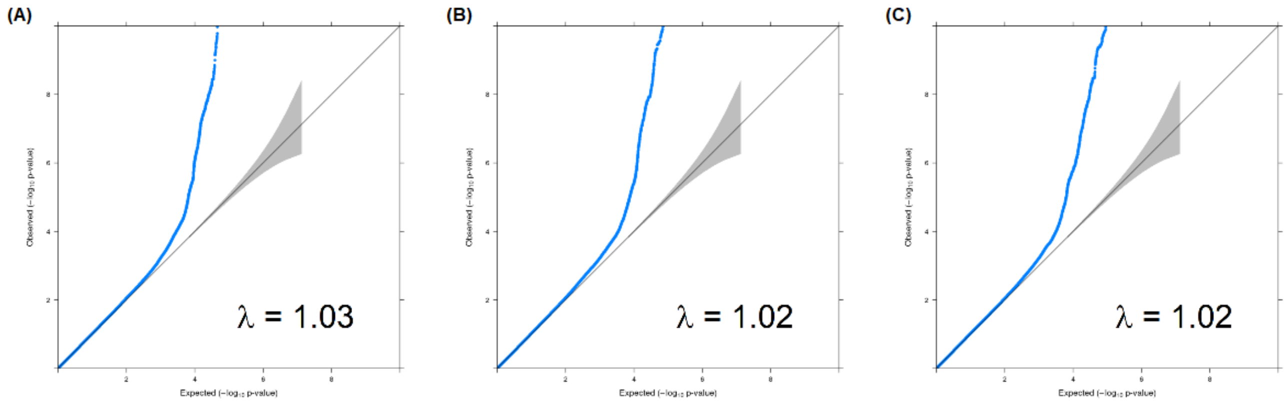

**Figure S2**: QQ plots along with genomic inflation factors ($\lambda$) for the 1 df test of GxE interaction across three maternal exposures from meta-analysis of GENEVA and POFC studies shown in Figure 3. (A) QQ plot for 1 df test of GxSmoking interaction; (B) QQ plot for 1 df test of GxAlcohol interaction; (C) QQ plot for 1 df test of GxVitamin interaction.

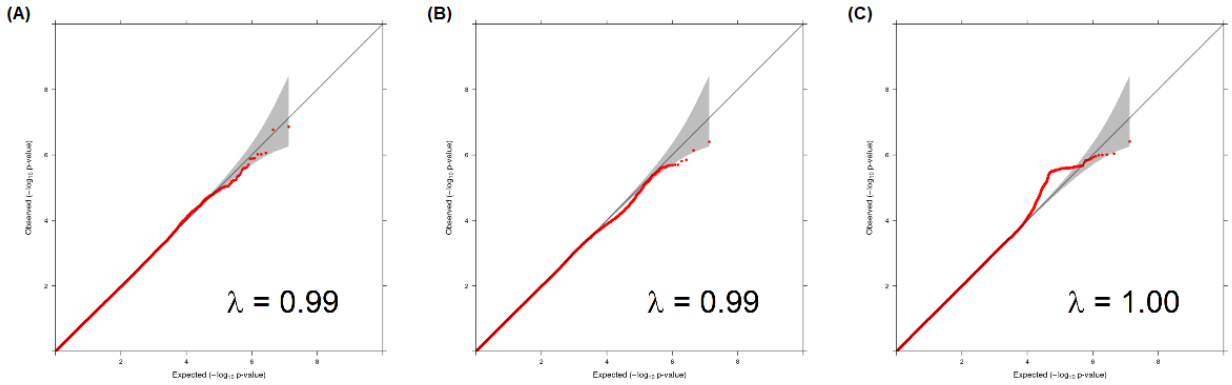

**Figure S3**: Region around *ANTXR1* which yielded suggestive evidence of GxVitamin interaction in meta-analysis of GENEVA and POFC in the 1 df test. SNP rs68079474 had a low frequency in all racial/ethnic groups (0.08 among people of European ancestry, 0.04 among parents of Asian ancestry, and 0.12 among Latin Americans).

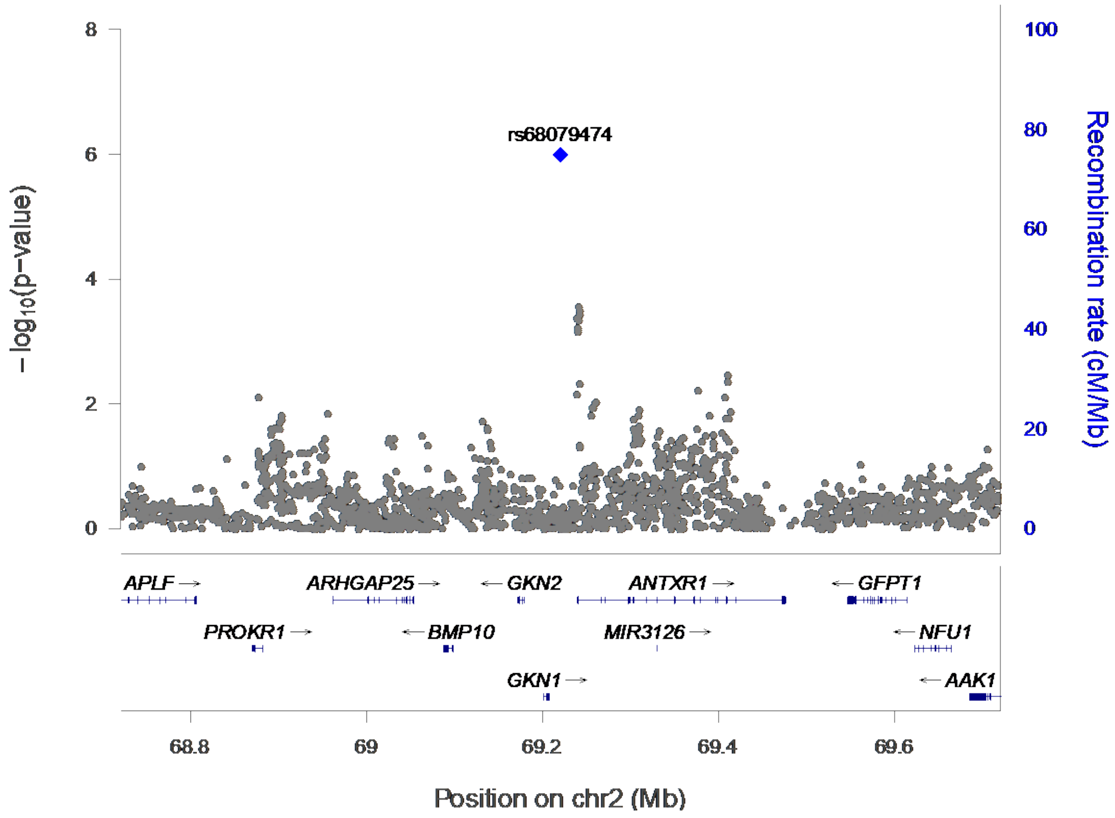

Supplement: Supplementary file 1 [file Data_Sheet_1.PDF]
